# Impact and Risk of Moral Injury Among Deployed Veterans: Implications for Veterans and Mental Health

**DOI:** 10.3389/fpsyt.2022.899084

**Published:** 2022-06-06

**Authors:** Joseph A. Boscarino, Richard E. Adams, Tiah J. Wingate, Joseph J. Boscarino, Thomas G. Urosevich, Stuart N. Hoffman, H. Lester Kirchner, Charles R. Figley, William P. Nash

**Affiliations:** ^1^Department of Population Health Sciences, Geisinger Clinic, Danville, PA, United States; ^2^Department of Sociology, Kent State University, Kent, OH, United States; ^3^Department of Neurosurgery and Brain Repair, University of South Florida, Tampa, FL, United States; ^4^Ophthalmology Service, Geisinger Clinic, Mount Pocono, PA, United States; ^5^Department of Sleep Medicine, Geisinger Clinic, Danville, PA, United States; ^6^School of Social Work, Tulane University, New Orleans, LA, United States; ^7^Department of Veterans Affairs (VA), Greater Los Angeles Healthcare System, Los Angeles, CA, United States

**Keywords:** veterans, moral injury, PTSD, depression, pain, substance misuse, fear of death, unit support

## Abstract

The impact of “moral injury” (MI) among deployed veterans, defined as actions in combat that violate a veteran's moral beliefs and result in psychological distress, has increasingly become a significant clinical concern separate from other trauma- and stressor-related disorders. MI involves severe distress over violations of core beliefs often followed by feelings of guilt and conflict and is common among veterans with PTSD. While the psychological impact of PTSD is well-documented among veterans, this has been done less so with respect to MI. We studied MI among 1,032 deployed veterans who were outpatients in a large non-profit multi-hospital system in central Pennsylvania. The study included active duty and Guard/Reserve members, as well as veterans who were not Department of Veterans Affairs (VA) service users. Our hypothesis was that, controlling for other risk factors, veterans with high MI would have current mental disorders. Our secondary hypothesis was that MI would be associated with other psychopathologies, including chronic pain, sleep disorders, fear of death, anomie, and use of alcohol/drugs to cope post deployment. Most veterans studied were deployed to Vietnam (64.1%), while others were deployed to post-Vietnam conflicts in Iraq and Afghanistan and elsewhere. Altogether, 95.1% of the veterans were male and their mean age was 61.6 years (SD = 11.8). Among the veterans, 24.4% had high combat exposure, 10.9% had PTSD, 19.8% had major depressive disorder, and 11.7% had a history of suicidal thoughts. Based on the Moral Injury Events Scale (MIES), 25.8% had high MI post deployment, defined as a score above the 75th percentile. Results show that high MI among veterans was associated with current global mental health severity and recent mental health service use, but not suicidal thoughts. In addition, as hypothesized, MI was also associated with pain, sleep disorders, fear of death, anomie, use of alcohol/drugs to cope post-deployment, and poor unit support/morale during deployment. Deployed veterans with MI are more likely to have current mental health disorders and other psychological problems years after deployment. Further research is advised related to the screening, assessment, treatment, and prevention of MI among veterans and others after trauma exposures.

## Introduction

In the current study, we examine the impact of “moral injury” (MI) among veterans, defined as the consequences of warzone exposures that violate the veteran's moral beliefs and result in severe psychological distress (1, 2). Consistent with earlier research (3), the objective of this study is to assess the post-deployment impact of moral injury among veterans, controlling for pre-deployment, deployment, and post-deployment risk factors. Given previous work (4, 5), our hypothesis was that moral injury would be associated with warzone exposures and the onset of mental health disorders and other psychopathology post-deployment. To assess this, we used a prediction models employed in past research (6–8). Our primary hypothesis is that MI would be associated with current mental health disorders, suicidal thoughts, mental health treatment, low self-esteem, among other risk factors, such as low unit deployment support (3, 7–10).

Our sample includes veterans from different conflicts recruited from non-VA community hospitals and include active-duty, Guard/Reserve, as well as former service members (3, 6, 8–10). We expected that veterans would have both risk and protective factors related to moral injury, including level of support, service history, among other factors (11, 12). This study is needed, since policy changes in the US have expanded access to care for veterans outside of the VA system (13). Understanding deployment-related risk factors among veterans is imperative for effective prevention and treatment of mental health disorders among veterans (6).

We note that while the concept of moral injury is still evolving, the traditional military model, the “combat stress” model, is that combat stress reactions occur following significant combat exposures, being taken prisoner of war, and/or following battlefield abandonment of service members (4). These experiences are often followed by feelings of guilt, despair, and in some cases character deterioration (1). Following the Vietnam War, however, the focus was on a trauma psychology model, which emphasized the impact of traumatic exposures, the onset and treatment of fear reactions, and emergence of a traumatic stress model (6, 9, 10). The traditional military model has been mostly focused management of psychological casualties in combat and the return of service members to active duty (4). The trauma model is grounded in psychoanalytic approach and is based on fear extinction and has focused on post deployment interventions, however, both models have merit. In addition, the earlier MI research focused on active-duty military personnel and VA patients using limited research measures (7). In the current study we used a valid and reliable MI scale (5), together with contemporary psychiatric instruments to assess MI among a large, diverse sample of veterans seen in community hospitals, where most veterans receive care (14, 15). Furthermore, research suggests moral injury may complicate PTSD treatment when present comorbidly, which is a clinical challenge (16, 17). Addressing MI among veterans is important because failing to treat this condition may cause greater harm post-deployment (1). Since the service branches typically support chaplaincy services, as do VA and Non-VA hospitals, this might be a practical yet effective intervention avenue if faith orientation is entangled in the moral conflict. While there is discussion on how best to assess MI (5), it clear that MI is a complex, multifactorial phenomenon associated with warzone exposures and clinicians should consider multiple resources to address this issue, including spiritual interventions, if needed. In addition, while our primary hypothesis is that MI is associated with current stress-related mental health disorders, we note that, based on past research (3, 6, 8, 9, 14), our secondary hypothesis is that MI will also be associated with a broad range of other psychopathologies, including sleep, substance misuse, pain, and neurotic disorders (9).

## Materials and Methods

### Study Sample

The population for the current study included a random sample of US military veterans (6, 8, 18). All veterans in the study were outpatients in the Geisinger Clinic, the largest private multi-hospital system in central Pennsylvania (3). In 2007, Geisinger initiated a registry for veterans receiving care at outpatient sites in Pennsylvania. Over 35,000 patients provided this information, and these data were used to select a random sample of veterans for the current study. Geisinger is an integrated health services organization that serves more than 3 million residents throughout 45 counties in central, south-central, and northeast Pennsylvania. This area encompasses a 40,000 square kilometer (25,000 square mile) service area (see: www.geisinger.org). The Geisinger system includes approximately 30,000 employees, 1,600 employed physicians, 10 hospital campuses, a 551,000-member managed care plan, a medical school, and is an open healthcare system that accepts all payor types, including private payors, public insurance (Medicare, Medicaid), Tricare, as well as payments for VA care.

Following patient consent, trained interviewers administered structured health interviews by telephone from February 2016 through February 2017, using a computer-assisted telephone interviewing (CATI) system by Sawtooth Technology (Northbrook Illinois, USA). During the survey, trained mental health counselors were available for the veterans, if required. All veterans for the current study had one or more warzone deployments. Veteran status and deployment history were confirmed based on military records. Among the veterans identified for the surveys, all were under 76 years old and served in Vietnam or a post-Vietnam conflict (i.e., Iraq, Afghanistan, Persian Gulf, or other recent conflict). After a total 10 telephone call attempts for each identified study veteran, we were able to complete interviews with 55% of eligible veterans in the study (3). Using demographic data in the electronic medical record, the only significant differences found between survey responders and non-responders were that responders tended to be younger and more often married (3).

### Study Measures

#### Moral Injury

To assess MI, we used the Moral Injury Events Scale (MIES) (5). The MIES is a validated scale with high reported internal validity (Cronbach's alpha = 0.90), concurrent validity, and discriminant validity (5). The MIES has nine items scored on a 6-point Likert scale (coded from “strongly agree” to “strongly disagree”), and included items such as, “I saw things that were morally wrong,” “I am troubled by having witnessed others' immoral acts.” Factor analyses revealed that the scale included two factors, explaining 64% of the common variance: perceived transgressions and perceived betrayals. In the current study, we combined this 9-item scale by summing each participant's responses to the item, with higher scores reflecting higher MI levels. The MI measure had an inter-quartile range of 3–19 (median = 10). Given the scale's skewed distribution, linear models, such as analysis of variance, are limited (19). Thus, we coded the MI scale both as a dichotomous variable classified as high vs, low MI, based on the 75th percentile and as an ordinal-level variable, coded in quintiles, representing low, moderate, high, very high and highest categories (19). For the dichotomous MI measure, we used logistic regression and for the ordinal-level variable we used ordinal logistic regression. These logistic regression models also included other independent variables as covariates, together with the stress exposures and personality measures noted (19). Goodness of fit statistics are also provided for all logistic regression models, including the area under the ROC curve, the Hosmer-Lemeshow χ2 statistic, and the McFadden Test (19).

#### Mental Health Measures

Our study also included several mental health measures as dependent variables in estimating the association between MI and mental health status: These included the BSI-Anxiety and BSI-Global Severity scales, current depression, recent use of mental health services, and a single-item suicide ideation measure from the BSI (“Had thoughts of ending your life?”). The BSI-18 scales are based on self-report in the past 30 days and has been widely used in psychiatric research (20). The BSI scales are normalized and use a standardized score of 65 to define a case (21). The reliability and validity of these BSI scales are good (20–22). To assess PTSD, we used an instrument based on the *Diagnostic and Statistical Manual of Mental Disorder, Fifth Edition* (DSM-5), the PTSD Checklist for DSM-5 (23, 24). To receive a diagnosis of current PTSD, veterans had to meet the DSM-5 diagnostic criteria A through G. This PTSD scale has been used in several recent studies (25, 26) and is reported to be a valid and reliable PTSD scale (8) (Cronbach's alpha = 0.92).

Depression was assessed using a major depressive disorder scale based on the DSM-IV diagnostic criteria (27), which has been used in previous survey studies, including the National Woman's Study (NWS) among others (10, 28, 29) Data related to the validity of this depression scale were previously reported and suggest that this scale can be used to diagnose depression in community-based population studies (30) (Cronbach's alpha = 0.90). To meet criteria in the study, subjects had to meet the full criteria for major depressive disorder.

Use of mental health services was based on survey questions that asked about mental health treatments received in the past 12 months from mental health professionals, such as a psychiatrist, psychologist, social worker, mental health counselor, minister, etc., for problems with emotions or nerves or the use of alcohol or drugs. These mental health service questions were used and validated in past mental health research among both veterans and adult non-veterans (10, 20, 30).

#### Combat Exposure, Trauma Measures, Adverse Childhood Events

Combat exposure was based on the Combat Experience Scale and versions of this measure have been used in studies since the Vietnam War (31–33). (Cronbach's alpha = 0.81). Based on previous research, scale measures for combat exposure were divided into standard cut-points (e.g., high vs. not high) that were used in previous research described elsewhere (3). Our study also assessed the occurrence of lifetime traumatic events using a traumatic event scale (e.g., ever experienced forced sexual contact, domestic abuse, a serious accident, major a disaster, etc.) used in previous research (20, 34). As we had no measure to judge the severity of these events, we collapsed these into three categories: <3 traumatic events, 3–5 events, and six or more events, as noted elsewhere (29). A total of 21% of respondents experienced six or more lifetime traumatic events in the current study and we used this to define “high” traumatic exposure as described in previous research (29, 30). Nearly 80% of the veterans in the current study reported that the most significant lifetime stressor they experienced was warzone exposure (3). This traumatic event scale was developed from other trauma studies, was also used in previous research, and had good reliability and validity (20, 29, 34). The study also included a measure of Adverse Childhood Events (ACE), a scale widely used in health research (35, 36). As done in previous research, we used a percentile cut-point to define a case (10).

#### Social Support, Life Stressors, Self-Esteem, Wellbeing Measures

We also assessed unit support, social support, homecoming support, self-esteem, and current life stressors, as these could have an impact on current health status (i.e., confounded with our independent and dependent variables) (3, 8, 37, 38). Social support, homecoming support, and current life stressors scales were based on previous research and had excellent reported reliability and validity (8, 37, 39). Additionally, in the survey we assessed current reported pain, reported health status in the past 12 months. In the survey, we also assessed “fear of death” (“afraid of news of death,” afraid you may die soon,” etc.) and “anomie” (i.e., social alienation, normlessness) (“a person does not know who to trust,” “the situation of the average person is getting worse,” etc.) both scales were coded “high” vs. “low” based on quartiles used in past research (Cronbach's alpha = 0.87 and 0.70, respectively) (40, 41). Additionally, we assessed the use of alcohol or drugs to cope since deployment based on the Brief Coping Scale, a widely used and validated instrument (10).

#### Substance Misuse, Personality Measures

Furthermore, we included a measure of alcohol misuse using the CAGE scale, which assess alcohol dependence in the past 12 months. The CAGE scale is a widely used and validated measure of alcohol dependence symptoms (18, 42). Those who reported two or more symptoms in the past 12 months (e.g., “thought should cut down on drinking,” “criticized about drinking,” etc.) were classified as having probable alcohol dependence. Similarly, among those taking prescription opioids for pain in the past year, we assessed these patients for opioid use disorder using the Severity of Dependence Scale (SDS), a valid and reliable instatement used past research (43). We also assessed lifetime marijuana use (never used, some use, moderate use, heavy use), based previous research (43–45). Furthermore, our study included a valid measure of neurotic personality traits used in previous research (46), as well as a measure of antisocial personality (e.g., as a teenager, ever ran away from home, stole things, fought with parents, teachers, etc.) (47). Finally, our study included a survey measure of unit support/morale (categorized as high vs, low) that inquired about unit camaraderie, trust of others, leadership, and support during deployment based on the Deployment Risk and Resilience Inventory (Cronbach's alpha = 0.78) (48).

Given the predictors assessed, we based our analyses on previous conceptual models and empirical results and selected variables that reflected key demographics, military, stressful events, and personality factors (8, 49). We note that the main health outcomes assessed were based on the past 12 months (PTSD, depression, mental health service use, pain, sleep problems, etc.). However, the BSI scales used for anxiety, global severity, and suicidal thoughts were based on the past 30 days. We also note that the time fame used for the MI scale was based “*since joining the military*” and the timeframe for using alcohol/drugs to cope was based on “*since deployment*.”

#### Study Covariates/Control Variables

Independent and study control variables in this research included age, gender, race, warzone deployments, being drafted, concussion history, Guard/Reserve status, and combat exposure, which were derived from the study survey measures (10, 14). Warzone exposures included the Vietnam War, Persian Gulf War, Afghanistan/Iraq War, and “other” recent warzone deployments, as defined by the VA. Global War on Terrorism (GWOT) veterans were combined with Iraq/Afghanistan veterans, since these deployments were during the same timeframe and were in supporting theaters of operations.

Moral Injury likely involves survivor guilt and other concerns, including spiritual and conceivably existential issues (50–54). Consequently, we would expect the psychosocial impact of MI to be long-lasting resulting in enduing personality, relationship, and self-concept changes (9, 54, 55).

### Data Analyses

Our statistical analyses included descriptive statistics depicting the study population and we present the characteristics of the total population for the current survey (*N* = 1,032) and we show these results in Table 1. Due to the questionnaire length and study timing, we split the interviews into two surveys, the original baseline survey and a second survey about 7–8 months later (*N* = 1,032) that included additional scales, based on veteran feedback and exploratory analyses related to potential MI psychopathology. We begin by presenting descriptive statistics and cross-tabulation results showing the association between high vs. low MI and both our independent and study control variables (Table 1). However, to assess the overall contribution of these measures in predicting post-deployment MI, we conducted logistic multivariate analyses (MVA) regressions using the stress, trauma, and symptom measures described to predict high vs. low MI (Table 2). In Table 3, we present the multivariate results for the health main health outcomes of interest, with MI score used as an ordinal quintile-level variable (scored 0–4; *n* = 202, *n* = 210, *n* = 205, *n* = 202, *n* = 213, respectively) in a stepwise logistic regression predicting current mental health status (56). To select these predictor variables, we used stepwise backwards logistic regression (57, 58), and compare these results to the stress process conceptual model in selecting candidate variables (3).

**Table 1:** High moral injury score vs. low score by study independent/control variables, based on Col. % (*N* = 1,032).

**Variable**	**Study variable (totals) [*n* (%)]**	**Moral injury 75th percentile or higher**		**OR**	***P =***
		**Yes (%)**	**No (%)**		
Age (M=61.6, SD=11.8)	65+ [670 (65.0)]	190 (71.4)	480 (62.8)	1.48	0.011
	18–64 [360 (35.0)]	76 (28.1)	284 (36.2)	1.00	–
Guard/reserve	Yes [373 (36.1)]	79 (29.6)	294 (38.4)	0.67	0.009
	No [659 (63.9)]	188 (70.4)	471 (61.6)	1.00	–
Combat high	Yes [252 (24.4)]	103 (38.6)	149 (19.5)	2.56	<0.001
	No [780 (75.6)]	164 (61.4)	616 (80.5)	1.00	–
Drafted	Yes [210 (20.3)]	70 (26.2)	140 (18.3)	1.59	0.006
	No [822 (79.7)]	197 (73.8)	625 (81.7)	1.00	–
Vietnam war deployment	Yes [661 (64.1)]	190 (71.2)	471 (61.6)	1.54	0.004
	No [371 (35.9)]	77 (28.8)	294 (38.4)	1.00	–
Low unit support/morale	Yes [211(20.4)]	88 (33.0)	123 (16.1)	2.57	<0.001
	No [821 (79.6)]	179 (67.0)	642 (83.9)	1.00	–
High stress past year	Yes [209 (20.3)]	83 (31.1)	126 (16.5)	2.29	<0.001
	No [823 (79.7)]	179 (67.0)	642 (83.9)	1.00	–
High lifetime trauma	Yes [207 (20.1)]	75 (28.1)	132 (17.3)	1.83	<0.001
	No [825 (79.9)]	192 (71.9)	633 (82.7)	1.00	–
Low homecoming support	Yes [298 (28.9)]	114 (42.7)	184 (24.1)	2.35	<0.001
	No [734 (71.1)]	153 (59.8)	581 (74.5)	1.00	–
High child abuse/neglect	Yes [159 (15.4)]	56 (21.0)	103 (13.5)	1.71	0.004
	No [873 (84.6)]	211 (79.0)	662 (86.5)	1.00	–
High fear of death	High [273 (26.5)]	109 (40.8)	164 (21.4)	2.53	<0.001
	Low [759 (73.5)]	158 (59.2)	601 (78.6)	1.00	–
High neuroticism	Yes [466 (45.2)]	163 (61.0)	303 (39.6)	2.39	<0.001
	No [566 (54.8)]	104 (39.0)	462 (60.4)	1.00	–
Low self–esteem	Yes [222 (21.5)]	104 (39.0)	118 (15.4)	3.50	<0.001
	No [810(78.5)]	163 (20.1)	647 (84.6)	1.00	-
Repression to cope	Yes [289 (28.0)]	118 (44.2)	171 (22.4)	2.95	<0.001
	No [743 (72.0)]	149 (55.8)	594 (77.6)	1.00	–
High anomie	High [274 (26.6)]	120 (44.9)	154 (20.1)	3.24	<0.001
	Low [758 (73.4)]	147 (55.1)	611 (79.9)	1.00	–
Cage positive alcohol score	Yes [64 (6.2)]	28 (10.5)	36 (4.7)	3.37	0.001
	No [968 (93.8)]	239 (89.5)	729 (95.3)	1.00	–
Used alc./drugs to cope	Yes [157 (15.2)]	72 (27.0)	85 (11.1)	2.95	<0.001
	No [875 (84.8)]	195 (73.0)	680 (88.9)	1.00	–
Anti-social personality	Yes [271 (23.6)]	94 (35.2)	177 (23.1)	1.81	<0.001
	No [761 (73.7)]	173 (64.8)	588 (76.9)	1.00	–
Concussion history	Yes [291 (28.2)]	110 (41.2)	181 (23.7)	2.26	<0.001
	No [741 (71.8)	157 (58.8)	584(76.3)	1.00	–
Pain interferes	Yes [357 (34.6)]	118 (44.2)	239 (31.2)	1.74	<0.001
	No [675 (65.4)]	149 (55.8)	526 (68.8)	1.00	–
Sleep problems	Yes [582 (56.4)	182(68.3)	400 (52.3)	1.95	<0.001
	No [450 (43.6)]	85 (31.8)	365 (47.7)	1.00	–
Ever used marijuana 50+ times	Yes [95 (9.2)]	38 (14.2)	57 (7.5)	2.06	0.002
	No [937 (90.8)]	229 (85.8)	708 (92.5)	1.00	–
Mental health treat. past year	Yes [233 (22.6)]	105 (39.3)	128 (16.7)	3.23	<0.001
	No [799 (77.4)]	162 (60.7)	637 (83.3)	1.00	–
Opioid dependence past year	Yes [46 (4.5)]	20 (43.5)	247 (25.1)	2.30	0.008
	No [986 (95.5)]	26 (56.5)	739 (74.9)	1.00	–
Column % =		(26%)	(74%)		

**Table 2:** Stepwise multivariable logistic regression predicting high vs. low MI score (*N* = 1,029)^‡^.

**Variables**	**OR^**†**^**	***z***	**95% CI**	***p*-value**
Age (years)*	0.98	−1.30	0.96–1.01	0.193
Female sex*	0.97	−0.08	0.41–2.25	0.935
High neuroticism	1.46	2.24	1.05–2.02	0.025
High combat exposure	2.01	3.97	1.42–2.84	0.001
Low self-esteem	2.07	3.90	1.43–2.96	<0.001
Used alc./drugs to cope	1.57	2.16	1.04–2.37	0.031
Vietnam war service	2.31	2.56	1.22–4.38	0.010
Low unit deploy. support	2.04	3.86	1.42–2.92	<0.001
High fear of death	1.75	3.22	1.24–2.46	0.001
High anomie	2.21	4.63	1.58–3.09	<0.001
Antisocial disorder	1.45	2.11	1.03–2.05	0.035
Lifetime marijuana use**	1.27	1.98	1.00–1.61	0.047
High repressive coping	1.48	2.20	1.04–2.11	0.028

*Odds Ratio.*

*Age and Sex forced into to the model at the first step. Results based on backwards stepwise elimination.

**For logistic regression marijuana use was coded as an ordinal variable classified as: never used, ever used occasionally, but <50 times, and ever used 50 or more times.

*^‡^Area under ROC = 0.78; Hosmer-Lemeshow χ^2^ = 7.93, df = 8, p = 0.440*.

**Table 3:** Multivariable logistic regression odds ratios and *p*-values predicting PTSD, depression, anxiety, global severity, suicidal thoughts, and mental Health treatment among veterans from selected neuro-psychosocial predictor variables.

**Variables**	**Ever PTSD = 10.9% OR, *P*-value**	**Variables**	**Ever depression = 19.8% OR, *P*-value**	**Variables**	**Current anxiety = 9.7% OR, *P*-value**	**Variables**	**Current global severity = 10.0% OR, *P*-value**	**Variables**	**Recent suicidal thoughts = 5.1% OR, *P*-value**	**Variables**	**Recent mental health treat. = 22.6% OR, *P*-value**
Age (years)	0.96, 0.003	Anomie	2.03, 0.017	Low resil.	4.08, <0.001	Low resilience	3.39, <0.001	Low resilience	4.17, <0.001	Sleep probs	1.53, 0.036
Cage positive	2.98, 0.012	Low resilience	3.54, <0.001	Neuroticism	3.28, <0.001	Sleep probs	2.97, 0.003	Pain interferes	2.23, 0.011	Female sex	3.21, 0.004
Low resilience	2.98, 0.002	Neuroticism	3.11, 0.002	High stress	2.22, 0.002	Neuroticism	2.65, 0.001	Neuroticism	2.07, 0.038	High repression	1.82, 0.002
Neuroticism	5.50, <0.001	High combat	2.01, 0.019	High combat	1.97, 0.010	High stress	2.28, 0.002	Substances cope	3.34, <0.001	High combat	1.61, 0.020
Concussion Hx	3.46, <0.001	Sleep probs	4.28, 0.003	Sleep probs	2.62, 0.006	High repression	2.13, 0.005	Neglect/abuse	2.14, 0.022	Concussion Hx	2.50, <0.001
High antisocial	0.49, 0.042	Vietnam serv.	0.36, 0.001	Neglect/abuse	2.16, 0.006	Neglect/abuse	2.16, 0.007			Neuroticism	2.15, <0.001
Sleep probs	4.96, 0.002	High repression	1.81, 0.048	High repress.	2.63, <0.001	Substance coping	2.17, 0.005			Substance coping	2.94, <0.001
Pain interferes	2.83, 0.003	Pain interferes	2.14, 0.013	Pain interferes	2.87, <0.001	Pain interferes	4.87, <0.001			Pain interferes	1.65, 0.008
High repression	2.70, 0.002					MI Score^‡^	1.25, 0.028			Vietnam serv.	0.42. <0.001
High stress	3.15, <0.001									High stress	1.97, 0.001
										MI score^‡^	1.32, <0.001
AUC = 0.92^†^		AUC = 0.89^†^		AUC = 0.89^‡^		AOC = 0.92^‡^		AOC = 0.85^‡^		AOC = 0.84^‡^	
McFadden *R*^2^ = 0.40		McFadden *R*^2^ = 0.29		McFadden *R*^2^ = 0.35		McFadden *R*^2^ = 0.39		McFadden *R*^2^ = 0.21		McFadden *R*^2^ = 0.28	
*N* = 1,032		*N* = 1,032		*N* = 1,027		*N* = 1,021		*N* = 996		*N* = 1,032	
H-L test χ2=	6.12, *p* = 0.63*	H-L test χ2=	5.88, *p* = 0.66*	H-L χ2 test =	7.99, *p* = 0.43*	H-L χ2 test =	7.38, *p* = 0.50*	H-L χ2 test =	4.77, *p* = 0.57*	H-L χ2 test =	6.43, *p* = 60*

*Area under ROC curve.*

*H-L, Hosmer-Lemeshow χ2 Test.

*^‡^Coded as an ordinal scale based on quintile distribution of MI score: Low, moderate, High, Very high, and Highest*.

In the final regression models key risk/protective factors (e.g., lifetime trauma exposure, number of deployments, other stressors, and demographics) were entered, followed MI by including these variables in the regression model (Table 3). All variables in the final multivariable logistic models are included in the analyses shown in the table if they were <0.05 probability. However, due to the number of variables assessed in our study (see Table 1), we used a *p*-value <0.01 to define clinical significance in our study. Also, for brevity, we summarize the non-diagnostic symptom measures (e.g., anomie, pain, fear of death, sleep problems, anomie, etc.) in the results section showing the indicator variable with the more detailed (i.e., reference category) results are available from the study PI upon request (JAB). As a final check of our regression model, we also conducted an ordinal logistic MVA regression predicting MI as an original scale (coded none, low, moderate, very high, and highest) and describe these findings, with the detailed results provided upon request from the study PI (JAB). For predicting current PTSD, depression, and anxiety, we also assessed interaction effects for MI by recent mental health treatment and report these results (these results are available from the study PI, upon request). Finally, we used Cluster Analyses to confirm the symptoms identified in the regression models (59). We present these cluster results in Table 4. In the discussion section, we review these results as they relate to screening and care for veterans with possible MI. Statistical analyses were conducted using Stata, version 15.1 and SPSS, version 20 software (58, 60).

**Table 4:** Moral injury cluster analysis results using study predictor variables.

Study variables*	**Cluster one (*n* = 745)**	**Cluster two (*n* = 287)**
Age (in years)	68.22	44.56
High combat exposure vs. low	0.25	0.23
Vietnam veteran vs. Not Vietnam veteran	0.89	0.00
Low unit support during deployment vs. not low	0.21	0.19
High stressful life event past year vs. not high	0.16	0.32
High lifetime trauma vs. not high	0.17	0.29
Low homecoming support vs. not low	0.40	0.01
High childhood abuse/neglect vs. not high	0.15	0.17
High fear of death vs. not high	0.24	0.32
High neuroticism vs. not high	0.44	0.49
Low self-esteem vs. not low	0.20	0.26
Used repression to cope vs. not use	0.27	0.30
High anomie vs. not high	0.30	0.17
Used alcohol/drugs to cope post deployment vs. not used	0.14	0.17
Antisocial personality vs. no antisocial personality	0.23	0.34
Pain interfered a lot in past year vs. did not interfered	0.34	.0.36
Problems sleeping in past year vs. no problems	0.55	0.60
Ever had major depressive disorder vs. did not have	0.15	0.32
Had concussion during deployment vs. did not have	0.29	0.27

*Analyses stopped after five iterations and included 2 cluster using k-means method. With exception of age, all variables are binary, coded high vs. low.

### Institutional Review Board Approval

This study was approved by the Institutional Review Boards of the Geisinger Clinic (IRB # 2015-0441) and the US Department of Defense (IRB # A-18989). All patients provided their informed consent to participate in the study and were offered small monetary incentives for participation. The study data were also protected by a Certificate of Confidentiality (CoC) issued by the National Institutes of Health (NIH). *This study was conducted in accordance with the principals stated in the Helsinki Declaration*.

## Results

Most veterans surveyed were over 65 years old (65%) and male (95.5%). In addition, 64.1% served in Vietnam, with the remaining veterans having served in Iraq, Afghanistan, or the Global War on Terror (Table 1, column 2). In terms of potential mental health risk factors 37% served on multiple tour, 24.4% had high combat exposure, 20.1% had high lifetime trauma exposure, 20.3% had high life stress exposure in the past year, and 15.4% had a history childhood abuse/neglect (Table 1, column 2). An examination of the bivariate cross-tabulation results (Table 1, columns 5–6), suggest that high MI was associated with: being older (OR = 1.48, *p* = 0.011), having high combat exposure (OR = 2.56, *p* < 0.001), being drafted (OR = 1.59, *p* = 0.006), serving in Vietnam (OR = 1.54, *p* = 0.004), reporting low unit support during deployment (OR = 2.57, *p* < 0.001), having high life stress in past year (OR = 2.29, *p* < 0.001), having high lifetime trauma exposure (OR = 1.83, *p* < 0.001), reporting low homecoming support (OR = 2.35, *p* < 0.001), having a high fear of death (OR = 2.53, *p* < 0.001), having high neuroticism (OR = 2.39, *p* < 0.001), having low self-esteem (OR = 3.50, *p* < 0.001), using repression to cope post deployment (OR = 2.95, *p* < 0.001), having high anomie (OR = 3.24, *p* < 0.001), having alcohol dependence on the CAGE scale (OR = 3.37, *p* < 0.001), using alcohol/drugs to cope post deployment (OR = 2.95, *p* < 0.001), having opioid dependence on the SDS scale (OR = 2.30, *p* = 0.008), and having antisocial personality traits (OR = 1.81, *p* < 0.001). Conversely, serving in the National Guard/Reserve (OR = 0.67, *p* = 0.009) was related to lower MI scores. In addition, having an in-service concussion, current pain, sleep problems, and a history of heavy marijuana use were positively associated with having a high MI score (all *p*-values *p* <0.01) (Table 1, columns 5–6). Finally, having mental health treatment in the past year was also associated with high MI (OR = 3.23, *p* < 0.001).

Given these results, we also conducted a multivariable logistic regression predicting high vs. low MI score to discover the relative contributions of key variables and risk factors on MI symptom status. These results are shown in Table 2. As can be seen, several variables standout. These include low unit support/morale, high combat exposure, low self-esteem, high anomie, and fear of death (all *p*-values = or < than 0.01). The statistical fit for this regression appears is good, with an area under ROC = 0.78 and a Hosmer-Lemeshow χ2 test = 7.93, df = 8, *p* = 0.440 (Table 2). We note we also ran a logit prediction model with MI as a dependent ordinal variable (with MI coded as an ordinal variable: none, low, high, and very high) and the results were like Table 2, with a McFadden *R*^2^ = 0.094 [results available from study PI (JAB) upon request].

In multivariable analyses predicting mental health status [including, lifetime PTSD (10.9%), lifetime depression (19.8%), anxiety in the past month (9.7%), global mental health severity in the past month (10.0%), suicidal thoughts in the past month (5.1%), and mental health treatment in the past year (22.6%), are shown in (Table 3). Significant variables in these prediction models were low resilience, high neuroticism, sleep problems, pain, and high repression. We note that MI score in the MVA models was significant in predicting global severity and mental health treatment seeking with the other variables also in the model (Table 3). In addition, our cluster analyses results confirm these MVA model results (Table 4). As can be seen, in Table 4, Cluster One (*n* = 745) is associated with older veterans, Vietnam service, low homecoming support, but higher anomie. Conversely, Cluster Two (*n* = 287) is associated with younger veterans and having history of depression but is otherwise like Cluster One in terms of high neuroticism, low self-esteem, high repression, and current pain (Table 4).

## Discussion

Consistent with previous research (61), our hypothesis was that moral injuries among veterans would be associated with stress exposures, negative life events, and warzone exposure factors (8). As suggested, MI is a psychosocial construct, often involving fear, guilt, and for some spiritual issues (50–52). Consequently, we would expect the impact of this injury would be clinically significant, prompting the need for surveillance and ongoing research. This conclusion is supported by our results. MI is not only related to service experiences but also to interpersonal (e.g., low unit morale/support), and psychological factors (e.g., low self-esteem, high anomie, and higher fear of death). These associations are confirmed in the multivariate analysis predicting MI (Table 2), as well as in the Cluster Analysis (Table 4). The logistic regression models also suggest that high MI may be implicated in a variety of health outcomes. For example, higher MI was associated with global mental health severity, recent mental health service use, and substance misuse (Tables 2, 3). In summary, we found risk for MI among a community-based sample of veterans, including not only current mental health disorders, but the presence of a spectrum of psychopathology years after warzone deployments, including substance misuse. These variables may be important in identifying and screening veterans at risk for MI in different settings in the future. However, as suggested, no interaction effects were detected for recent treatments outcomes by MI, which is a good thing (7).

The current study has several strengths. First, we recruited a large random sample of community-based veterans. Second, we used validated scales and survey measures from previous research (3). Third, we included veterans from Vietnam through to current conflicts in Iraq and Afghanistan, something not typically done in veteran studies, but may be more representative of veterans overall (10). Fourth, we examined key post-deployment mental health outcomes and multiple risk factors using a valid and reliable scale furthering the utility of our MI scale.

Our study has several limitations, including that it was based on a cross-sectional survey. Because of this limitation, it is possible that some associations found in could be reversed (62), such that those with post-deployment mental health problems may have a more negative recall of different symptoms and other health-related outcomes. In addition, although our study was based on a large survey, the study was conducted among mostly Caucasian patients in a multi-hospital system located in central and northeastern Pennsylvania. Furthermore, we found some survey participation differences, whereby survey respondents tended to be younger and more often married (both *p*-values < 0.05), compared to non-respondents (10). This limitation may also apply to the second survey as well, since only 60% of veterans complete the latter. Thus, it may not be possible to generalize these findings to other study populations (62). As noted elsewhere, however, there are few robust samples of veterans available for research, since this population tends to be dynamic, given multiple deployments, ongoing conflicts, VA policy changes, fluctuations in service use, and the aging of the veteran population (3, 63, 64). In addition, most veterans do not consistently use the VA system for health care (65), which complicates using representative samples of veterans for population health research. Furthermore, given the number of variables, assessed, multiple comparisons may be an issue in our study, although we raised the “clinical significance” level to *p* < 0.01 to avoid this problem. Finally, another limitation is that the main study measure in the current study, combined a 9-item scale that had an inter-quartile range of 3–19 (median = 10), suggesting the distribution of scores derived from this measure was skewed. Because of this limitation, we used this scale as a dichotomous variable and as an ordinal scale (coded as quintile-level variable from “low” to “highest” score, respectively) in stepwise logistic regressions predicting current mental health status (56). To select these predictor variables, we used stepwise backwards logistic regression (57, 58), and compared these results to the stress process conceptual model in selecting candidate variables (3), and then compared these results to our Cluster Analysis results, which were both similar.

## Conclusion

Despite these limitations, our findings are consistent with recent studies (6, 8). We suggest that services for returning veterans that target MI-specific risk factors post-deployment may result in better outcomes (7). The reasons why some veterans are at greater risk for MI is still unclear and may involve personality and moral value issues (18, 52), as well as factors related to sensory processing of stimuli (9, 66). As noted, MI is complicated and likely involves survivor's guilt and other issues related to perceived transgressions and perceived betrayals in combat (50, 51). Practical interventions could include mindfulness training, brief interventions, and other minimally invasive treatments, but further research is required (66–69). Adaptive disclosure (AD) is relatively recent intervention for active-duty service members (70). Noteworthy is that AD considers unique aspects of military service in war to address difficulties such as moral injury and traumatic loss that may not receive adequate attention by conventional treatments that primarily address fear-inducing experiences and their sequelae.

Moral injury is said to be a risk when there has been a betrayal of “what's right” either by a person in authority or by oneself, or in a high-stake situation (1). This can occur when a military unit is overrun forcing difficult choices to me made (1, 4). Clinical challenges in working with moral injury include coping with being made witness to atrocities and depravity through repeated exposure to trauma narratives, characteristic assignment of survivor's transference roles to clinicians, and the clinicians' countertransference emotions and judgments of self and others. A trustworthy clinical community and, particularly, a well-functioning clinical team provide protection for clinicians and are a major factor in successful outcomes with morally injured combat veterans (1).

However, following deployments, most veterans return to local communities and are typically seen in non-government facilities (71), complicating their psychological and emotional care. Therefore, providers in non-VA settings need to be aware of the potential impact moral injury and PTSD among veterans (15, 72), as well as the potential significance of “veteran identity” in the recovery process (61) The concept of moral injury may help clinicians gauge exposure to traumatic events that contradict the veterans' deeply held moral beliefs, which, may result in disordered thinking and mental illness among veterans' years afterwards. As we have shown, high combat exposure, history of other trauma exposures, high current life stressor, low self-esteem, and poor unit support/morale are also associated with moral injury. Furthermore, having a high fear of death, anomie, and neurotic personality traits are also associated with MI, as well as increased pain, sleep problems, and growing evidence of substance abuse involving misuse of both alcohol and drugs. Further research is warranted building from the current study and others. With the ending of the post-9/11 combat era and with state-sponsored terrorism diminishing, concurrent with a renewed focus on conventional warfighting, battlefield stress management, unit morale, and return of service members to their original units (4), may be the future emphasis in military combat medicine. Time will tell.

## Data Availability Statement

The datasets presented in this article are not readily available because these data are still being analyzed by the study team. Requests to access the datasets should be directed to JAB, jaboscarino@geisinger.edu; joseph.boscarino@gmail.com.

## Ethics Statement

This study was approved by the Institutional Review Boards of the Geisinger Clinic (IRB # 2015-0441) and the US Department of Defense (IRB # A-18989). This study was conducted in accordance with the principals stated in the Helsinki Declaration. The patients/participants provided their written informed consent to participate in this study.

## Author Contributions

JAB was responsible for study funding, research design, and drafting the first version of the manuscript. JJB, RA, and TW assisted with analysis and manuscript writing. HK provided statistical consulting. WN and CF provided clinical consultations related to mortal injury. All the authors reviewed and approved the manuscript.

## Funding

Funding was provided by: Geisinger Auxiliary Fund, the Kline & Ditty Health Fund (#752109 & #762170), National Institute of Mental Health (Grant No. R21-MH-086317), Wounded Warrior Project, and the Department of Defense (Contract No. W81XWH-15-1-0506) to JAB. The sponsors had no role in the analysis, writing, or submission of the manuscript for publication.

## Conflict of Interest

The authors declare that the research was conducted in the absence of any commercial or financial relationships that could be construed as a potential conflict of interest.

## Publisher's Note

All claims expressed in this article are solely those of the authors and do not necessarily represent those of their affiliated organizations, or those of the publisher, the editors and the reviewers. Any product that may be evaluated in this article, or claim that may be made by its manufacturer, is not guaranteed or endorsed by the publisher.
